# Altered expression of ADAR1, N4BP1, and PSME1 in PBMCs correlated with therapeutic outcomes in HBeAg-negative chronic hepatitis B patients treated with Peg-IFN-α

**DOI:** 10.3389/fcimb.2026.1749013

**Published:** 2026-04-13

**Authors:** Hao Pang, Xinglin Fu, Lüping Chen, Shuhan Yang, Fan Yang, Bo Qin

**Affiliations:** 1 Department of Infectious Diseases, Chongqing Key Laboratory of Infectious Diseases and Parasitic Diseases, The First Affiliated Hospital of Chongqing Medical University, Chongqing, China; 2 Central Laboratory, The First Affiliated Hospital of Chongqing Medical University, Chongqing, China

**Keywords:** ADAR1, hepatitis B virus, N4BP1, Peg-IFN-α, PSME1

## Abstract

**Background and aims:**

Pegylated interferon alpha (Peg-IFN-α) has the potential for eradicating hepatitis B surface antigen (HBsAg). The aim of our study is to investigate whether the expression levels of adenosine deaminase acting on RNA 1 (ADAR1), NEDD4-binding protein 1 (N4BP1), proteasome activator complex subunit 1 (PSME1) mRNAs in peripheral blood mononuclear cells (PBMCs) of HBeAg-negative chronic hepatitis B virus (HBV) patients are associated with the response to Peg-IFN-α treatment and HBsAg clearance.

**Methods:**

In this prospective study, HBeAg-negative chronic HBV patients treated with Peg-IFN-α were followed for 48 weeks. Patients were categorized into the virological response (VR) group and non-virological response (NVR) group based on the observed changes in HBV DNA and HBsAg levels at week 48 of treatment. Additionally, patients were classified into a serological response (SR) group and a non-serological response (NSR) group according to whether serum HBsAg loss or seroconversion occurred. The expression levels of ADAR1, N4BP1, and PSME1 mRNAs in PBMCs were detected by real-time quantitative PCR. The diagnostic performance of ADAR1, N4BP1, and PSME1 was assessed by analyzing the receiver operating characteristic (ROC) curve and calculating the area under the curve (AUC).

**Results:**

After the treatment period, the VR and SR rates were 47.25% and 35.16%, respectively. Dynamic changes in ADAR1, N4BP1, and PSME1 mRNA levels differed significantly between the VR and NVR groups, as well as between the SR and NSR groups. Multivariate analysis revealed that ADAR1 was independently associated with VR and SR at weeks 12 and 24; N4BP1 was independently associated with VR at weeks 12 and 24; PSME1 was independently associated with VR and SR at weeks 12 and 24. At week 24, the AUCs for ADAR1 in predicting VR and SR were 0.9230 and 0.8554. N4BP1 had AUCs of 0.7393 for VR at week 12 and 0.7198 for SR at week 24, while PSME1 had AUCs of 0.7418 for VR and 0.7426 for SR at week 12.

**Conclusions:**

ADAR1, N4BP1, and PSME1 are novel biomarkers for early therapeutic response to Peg-IFN-α and HBsAg clearance.

**Clinical Trial Registration:**

https://www.medicalresearch.org.cn/login, identifier 2023−311.

## Introduction

Chronic hepatitis B virus (HBV) infection represents a significant global health challenge, with the potential to progress to cirrhosis, decompensated liver cirrhosis, and an increased risk of hepatocellular carcinoma (HCC) (Yuen et al., 2018). To reduce the incidence of adverse liver clinical events, the treatment goal has evolved from achieving negativity for hepatitis B virus DNA (HBV DNA) and hepatitis B e antigen (HBeAg) to achieving negativity for hepatitis B surface antigen (HBsAg) (Terrault et al., 2018). The loss of HBsAg, with or without seroconversion to hepatitis B surface antibody (HBsAb), is considered a functional cure and represents the ideal endpoint of antiviral therapy for chronic hepatitis B (CHB) (Terrault et al., 2018). Current therapeutic options for chronic HBV infection include pegylated interferon alpha (Peg-IFN-α) and nucleoside analogues (NAs) (Lok, 2024). Compared to NAs, the HBsAg seroconversion rate among patients treated with Peg-IFN-α is significantly higher, although it remains relatively low (7.8%–10%) in the overall population (Bourlière et al., 2017; Farag et al., 2024). Previous studies have shown that HBsAg-negative patients have a markedly lower risk of developing HCC and cirrhosis compared to HBsAg-positive patients (Okada et al., 2024). Recent studies have indicated that patients with HBeAg-negative chronic HBV may either recover or progress to more severe stages of chronic hepatitis, potentially leading to end-stage liver disease (Chen et al., 2010; Chu and Liaw, 2007). Consequently, there is an urgent need for effective treatments and reliable predictions of therapeutic efficacy in this patient population.

Interferon alpha (IFN-α), a member of the type I interferons, was the first treatment approved for chronic hepatitis B, achieving a sustained response in a minority of patients, although it is associated with significant side effects (Zoulim and Durantel, 2015). Upon binding to its cognate receptor on the cell surface, IFN-α initiates a JAK/STAT signaling cascade, leading to the induction of over 300 interferon-stimulated genes (ISGs) (Sadler and Williams, 2008; Der et al., 1998). Numerous ISGs have been reported to inhibit HBV replication at various stages, primarily through transcriptional and post-transcriptional mechanisms (Robek et al., 2004; Uprichard et al., 2003; Zhao et al., 2024). For instance, STAT1, SMCHD1, and PML bind to the cccDNA minichromosome, establishing a suppressive epigenetic status for cccDNA (Cheng et al., 2020); TRIM5γ inhibits HBV transcription by promoting the degradation of HBx (Tan et al., 2019); and zinc finger antiviral protein (ZAP) and ISG20 bind to HBV RNA, accelerating its decay (Liu et al., 2017; Mao et al., 2013). Despite these advancements, reliable and definitive biomarkers for predicting treatment outcomes are currently lacking. Identifying such biomarkers is crucial, as they could offer valuable insights into the therapeutic efficacy of Peg-IFN-α prior to treatment initiation.

Adenosine deaminase acting on RNA-1 (ADAR1) is an interferon-inducible gene that catalyzes the deamination of adenosine to inosine, a process known as A-to-I RNA editing (Nishikura, 2010, 2016; Gatsiou et al., 2018). Recent studies have demonstrated that ADAR1 exerts an antiviral effect against HBV infection via enhancing the level of miRNA-122 in hepatocytes (Liu et al., 2019). NEDD4-binding protein 1 (N4BP1), an E3 ligase Itch inhibitor and a ubiquitin-binding endoribonuclease (Oberst et al., 2007), has been identified as a suppressor of cytokine production and is cleaved by caspase-8 (Gitlin et al., 2020). N4BP1 was identified as an ISG following its original discovery through high-throughput screening (Schoggins et al., 2011). One study has recently identified N4BP1 as exhibiting an anti-HBV effect, thereby establishing it as a novel host factor that counteracts HBV production by regulating the levels of 3.5 kb and 2.1/2.4 kb HBV RNA (Kobayashi et al., 2025). Proteasome activator complex subunit 1 (PSME1), also categorized as an ISG (Makjaroen et al., 2018; Shaw et al., 2017), plays a critical regulatory role in intracellular proteolytic pathways mediated by the proteasome (Ma et al., 1992). Another recent study found that PSME1 affects HBV replication through its interaction with HBc, suggesting that PSME1 may be a potential candidate for anti-HBV therapy (Liu et al., 2024). Despite the critical involvement of these ISGs in modulating the HBV life cycle, their clinical utility as predictive biomarkers remains largely unexplored. Most previous studies aimed at identifying biomarkers predictive of interferon efficacy have focused on classical ISGs, including MX, SOCS3, and TRIM14 (Han et al., 2017; Li et al., 2022). Although the predictive value of these genes has been extensively investigated in the context of interferon therapy for chronic hepatitis B, their performance has often been inconsistent across different patient cohorts. In contrast, these ISGs examined in the present study (namely ADAR1, N4BP1, and PSME1) modulate HBV replication through distinct mechanisms, thereby providing complementary coverage of key antiviral pathways activated by interferon signaling and offering a deeper biological rationale and broader prospects for clinical application. Notably, emerging evidence suggests their clinical relevance in liver disease. Specifically, ADAR1 expression has been linked to liver fibrosis progression and HCC prognosis (Medrano et al., 2017; Wang et al., 2024). Furthermore, N4BP1 expression is significantly elevated in HBV DNA-negative patients compared with HBV DNA-positive patients, with scRNA-seq analysis similarly demonstrating marked upregulation in patients achieving functional cure relative to CHB patients (Kobayashi et al., 2025). A recent proteomic study on the natural history of chronic HBV infection reported significantly elevated serum PSME1 levels in CHB patients, establishing its utility in distinguishing the natural history of chronic HBV infection (Xun et al., 2022). Importantly, despite their potential, no study has systematically investigated the dynamic changes in ADAR1, N4BP1, and PSME1 mRNA levels in peripheral blood mononuclear cells (PBMCs) during Peg-IFN-α therapy, nor their associations with treatment outcomes and their predictive value for interferon efficacy in HBeAg-negative CHB patients. Therefore, we seek to evaluate their potential as practical early biomarkers of interferon response in a real-world cohort of HBeAg-negative patients.

The aim of this study is to investigate the relationship between the mRNA levels of ADAR1, N4BP1, and PSME1 in PBMCs and the response to Peg-IFN-α treatment, as well as HBsAg clearance in HBeAg-negative chronic HBV patients. The identification of these biomarkers is expected to facilitate the development of individualized therapeutic strategies, thereby enabling clinicians to tailor management approaches to meet the specific needs of each patient.

## Methods

### Patient recruitment

This study was a prospective observational cohort study involving individuals diagnosed with CHB. The cohort included three groups: (1) 91 HBeAg-negative CHB patients receiving at least 48 weeks of Peg-IFN-α therapy, (2) 58 patients with untreated chronic HBV infection, and (3) 44 healthy controls (HC). The untreated CHB and healthy control groups served as baseline references to evaluate the impact of HBV infection on ISG expression in PBMCs prior to treatment. Sample size was calculated using G*Power (version 3.1.9.7). For comparisons between healthy controls and untreated CHB patients, a medium-to-large effect size (Cohen’s d = 0.65) and an allocation ratio of 0.60 were assumed to provide adequate power for detecting clinically meaningful differences in gene expression. With a two-sided α = 0.05 and a statistical power of 0.80, the minimum required total sample size was estimated to be 82 (HC = 31, untreated CHB = 51). To account for potential sample exclusion and ensure adequate statistical robustness, the planned enrollment for these groups was increased to 102 participants (44 HC and 58 untreated CHB). For treatment response analyses (responders vs. non-responders), a medium-to-large effect size (Cohen’s d = 0.65) and unequal allocation ratios of 0.80 and 0.50 were applied, reflecting the anticipated clinical response rates in real-world practice. Under these statistical assumptions (two-sided α = 0.05 and power = 0.80), the minimum required total sample sizes were estimated to be 78 and 86 for the virological and serological response comparisons, respectively. For within-group repeated-measures analyses across three time points (weeks 0, 12, and 24) in the treated group, a medium effect size (Cohen’s f = 0.30) was applied under the same α and power conditions, requiring at least 20 participants per subgroup. The final enrolled cohort exceeded the minimum sample size requirements for all planned analyses (**Supplementary Table 1**). A total of 91 HBeAg-negative patients, aged 18 to 65 years, were enrolled to receive a minimum of 48 weeks of Peg-IFN-α therapy at the Outpatient Infectious Diseases Department of The First Affiliated Hospital of Chongqing Medical University, between October 2023 and October 2024. Patients received Peg-IFN-α via subcutaneous injection at a dose of 180 μg once weekly. The study design is illustrated in a flowchart presented in **Supplementary Figure 1**. The diagnosis and management of HBeAg-negative CHB patients adhered to the 2018 guidelines issued by the American Association for the Study of Liver Diseases (AASLD) for the management of chronic hepatitis B infection (Terrault et al., 2018).

The inclusion criteria for this study were as follows: positive HBsAg, negative HBeAg, positive HBeAb, and positive HBcAb, a low HBV DNA load (< 2000 IU/mL), normal liver function, and no or mild liver inflammation or fibrosis. The exclusion criteria were: (1) co-infection with other hepatitis viruses, such as hepatitis C virus (HCV) or hepatitis D virus (HDV); (2) co-infection with other viral pathogens, including human immunodeficiency virus (HIV) and Epstein–Barr virus (EBV); (3) autoimmune liver disease; (4) severe liver damage or other liver diseases, such as alcoholic liver disease and fatty liver disease; (5) diagnosis of other malignant diseases; and (6) contraindications to interferon treatment.

All participants provided written informed consent. The research protocol received approval from the Ethics Committee of The First Affiliated Hospital of Chongqing Medical University (Reference number: 2023-311). For the assessment of treatment response, we evaluated virological and serological variables, including baseline and subsequent HBsAg titers measured every 12 weeks in HBeAg-negative CHB patients receiving Peg-IFN-α therapy. PBMCs were collected at baseline, 12 weeks, and 24 weeks, and the mRNA expression levels for intracellular ADAR1, N4BP1, and PSME1 were subsequently determined using qRT-PCR. Patients were categorized into virological response (VR) and non-virological response (NVR) groups based on changes observed in serum HBV DNA and HBsAg levels following 48 weeks of Peg-IFN-α treatment. A VR was defined as a decrease of > 2 log10 IU/mL in HBV DNA or a significant reduction in HBsAg levels (> 1 log10 IU/mL) or HBsAg clearance. Those who did not meet these criteria were classified as NVR. Serological response (SR) was defined as HBsAg seroconversion or HBsAg loss during treatment, while the absence of these serological changes was categorized as a non-serological response (NSR).

### Clinical and laboratory measurements

Quantitative real-time PCR with fluorescence detection was utilized to quantify HBV DNA levels, with a lower detection limit of 20 IU/mL (Roche Cobas TaqMan 48, Shanghai). The Abbott Architect i2000 system was employed for the serological assessment of HBsAg/HBsAb and HBeAg/HBeAb titers. Liver function parameters were determined via automated biochemical analysis using the Roche Cobas platform (Shanghai). The Fibrosis-4 (FIB-4) index was calculated for all participants using the formula: FIB-4 = [age (years) × AST (U/L)]/[platelet count (10^9^/L) × √ALT (U/L)]. Hematological profiles were obtained through automated hematology analyzers (Mairui BC-6600, Shanghai).

### RNA extraction and quantitative real-time PCR

A two-step quantitative reverse transcription polymerase chain reaction (qRT-PCR) was utilized to quantify the mRNA expression levels of ADAR1, N4BP1, and PSME1 in PBMCs. Peripheral venous blood samples (5 mL) were collected from all participants into EDTA-coated tubes. PBMCs were subsequently isolated within 4 hours of blood collection using density gradient centrifugation with Ficoll-Paque Plus (Cytiva, Sweden) according to the manufacturer’s protocol. Total RNA was extracted from PBMCs using TRIzol reagent (Invitrogen, Carlsbad, CA, USA). The quantity and purity of the extracted RNA were assessed using a spectrophotometer (NanoDrop 2000, Thermo Fisher Scientific, USA), ensuring that the A260/A280 ratio was between 1.8 and 2.0. This was followed by the synthesis of first-strand complementary DNA (cDNA) using a PrimeScript RT reagent Kit (Takara Bio, Kusatsu, Japan). Subsequently, real-time PCR amplification of the synthesized cDNA was performed using a Bio-Rad CFX96 fluorescence detection system (USA). The PCR conditions were as follows: pre-denaturation at 95 °C for 30 s, followed by 40 cycles of 95 °C for 5 s and 60 °C for 30 s, and a melting curve stage consisting of 95 °C for 5 s and 60 °C for 1 min, followed by cooling at 50 °C for 30 s (see the **Supplementary Methods** for details). Post-amplification melting curve analysis was routinely performed to confirm the specific amplification of a single target product and the absence of primer-dimers. Each qRT-PCR reaction was performed in a 20 μL volume containing 10 μL of TB Green Premix Ex Taq II (Takara Bio), 0.4 μL of each primer (10 μM), 2 μL of cDNA, and nuclease-free water. The expression levels of the target genes were assessed using GAPDH as the internal control, and relative mRNA quantification was calculated using the 2^-ΔΔCt method (Livak and Schmittgen, 2001). Briefly, the Ct value of each target gene was first normalized to the Ct value of GAPDH to obtain the ΔCt value. The ΔΔCt value was calculated by comparing each sample with the reference group, and the relative expression level was expressed as 2^-ΔΔCt. The mean Ct value from triplicate reactions was used for subsequent analyses. Reactions with a Ct standard deviation greater than 0.5 among triplicates were re-assayed to ensure data reliability. Detailed information regarding the primer sequences utilized in the quantitative PCR assays is presented in **Supplementary Table S2**.

### Statistical analysis

Statistical analyses and data visualizations were conducted using SPSS version 27.0 and GraphPad Prism version 10.1.2. Prior to comparative analyses, the normality of continuous variables was evaluated using the Shapiro–Wilk test, and serum concentrations of HBsAg and HBV DNA were logarithmically transformed. Continuous variables following a normal distribution are presented as mean ± standard deviation, while non-normal variables are reported as median with interquartile range (IQR). The independent samples t-test was applied to normally distributed data, and the Mann–Whitney U test was used for non-normal data. For the comparison of dynamic changes within the same patient cohort across multiple time points (weeks 0, 12, and 24), repeated-measures analysis of variance (ANOVA) was applied to normally distributed variables, whereas the Friedman test was used for non-normal variables. When significant differences were observed, *post-hoc* pairwise comparisons were performed with Bonferroni correction. Univariate and multivariate logistic regression analyses were performed to identify independent factors associated with VR and SR. Variables with P < 0.10 in univariate analyses or considered clinically relevant were entered into the multivariate models. Potential multicollinearity among predictors was assessed using the variance inflation factor (VIF), with a VIF < 5 indicating the absence of significant collinearity. Additionally, the goodness-of-fit of the multivariable logistic regression models was evaluated using the Hosmer–Lemeshow test, where a P-value > 0.05 indicated acceptable model calibration. The predictive performance of ADAR1, N4BP1, and PSME1 was assessed through receiver operating characteristic (ROC) curve analysis, with calculation of the area under the curve (AUC) and corresponding 95% confidence intervals (CIs). Optimal cutoff values were determined using Youden’s index (sensitivity + specificity − 1), with the threshold yielding the maximum index value. Differences between AUCs were evaluated with the DeLong test to determine statistical significance. All statistical tests were two-sided, with significance defined as P < 0.05.

## Results

### Baseline characteristics of all subjects and treatment response

All participants in this study were categorized into three distinct groups based on their disease status: (1) 58 patients with untreated CHB (27 males, 31 females), (2) 91 patients with HBeAg-negative CHB (49 males, 42 females) who were receiving Peg-IFN-α treatment, and (3) 44 healthy individuals (22 males, 22 females). Detailed baseline demographics and clinical characteristics are presented in **Table 1**. No significant statistical differences were observed between the treated and untreated CHB groups regarding HBsAg and HBV DNA levels. Furthermore, age, gender, ALT, AST, TBil, PLT, WBC levels, and the FIB-4 index showed no significant differences among all three groups. Among the 91 HBeAg-negative CHB patients who completed 48 weeks of Peg-IFN-α treatment, 32 (35.16%) exhibited a serological response, while 43 (47.25%) demonstrated a virological response (**Supplementary Tables S3, S4**). In terms of baseline HBV genotype distribution among these patients, 54 (59.3%) were infected with genotype B, 33 (36.3%) with genotype C, and 4 (4.4%) with other genotypes (**Supplementary Table S5**).

**Table 1:** Baseline characteristics in the groups.

Characteristics	HC	Untreated CHB	CHB treated with Peg-IFN-α	P value
Number (n)	44	58	91	
Age (year)	35.00 (24.75,45.00)	46.50 (37.00,50.25)	45.00 (34.00,49.00)	0.1452
Gender (male/female)	22/22	27/31	49/42	
HBsAg (log10 IU/mL)	UD	2.921 (2.143,3.304)	3.011 (2.068,3.419)	0.4642
HBV DNA (log10 IU/mL)	UD	1.699 (1.699,1.699)	1.787 (1.699,3.000)	0.1362
ALT (U/L)	26.09 ± 9.098	21.50 (15.75,37.50)	28.00 (18.00,39.00)	0.2059
AST (U/L)	24.50 (19.00, 35.00)	23.00 (20.00,35.00)	26.00 (20.00,36.00)	0.5697
WBC (×10^9/L)	5.925 ± 1.571	4.296 ± 1.317	4.429 ± 1.416	0.5610
TBil (μmol/L)	13.02 ± 3.804	11.05 (9.050,14.18)	10.90 ± 4.385	0.1612
PLT (×10^9/L)	187.3 ± 46.75	176.5 (135.8,191.8)	152.5 ± 50.31	0.0887
FIB-4 index	1.087 (0.732,1.494)	1.158 (0.365,1.585)	0.872 (0.576,1.321)	0.2380

UD, undetected; HC, healthy control; HBsAg, hepatitis B surface antigen; ALT, alanine aminotransferase; AST, aspartate aminotransferase; WBC, white blood cells; TBil, total bilirubin; PLT, platelet; FIB-4, the Fibrosis-4; The results are presented as the median and inter-quartile range or mean ± standard deviation; Bold value is statistically significant P < 0.05.

### ADAR1, N4BP1, and PSME1 mRNA levels in PBMCs are associated with HBV infection

**Figure 1:**
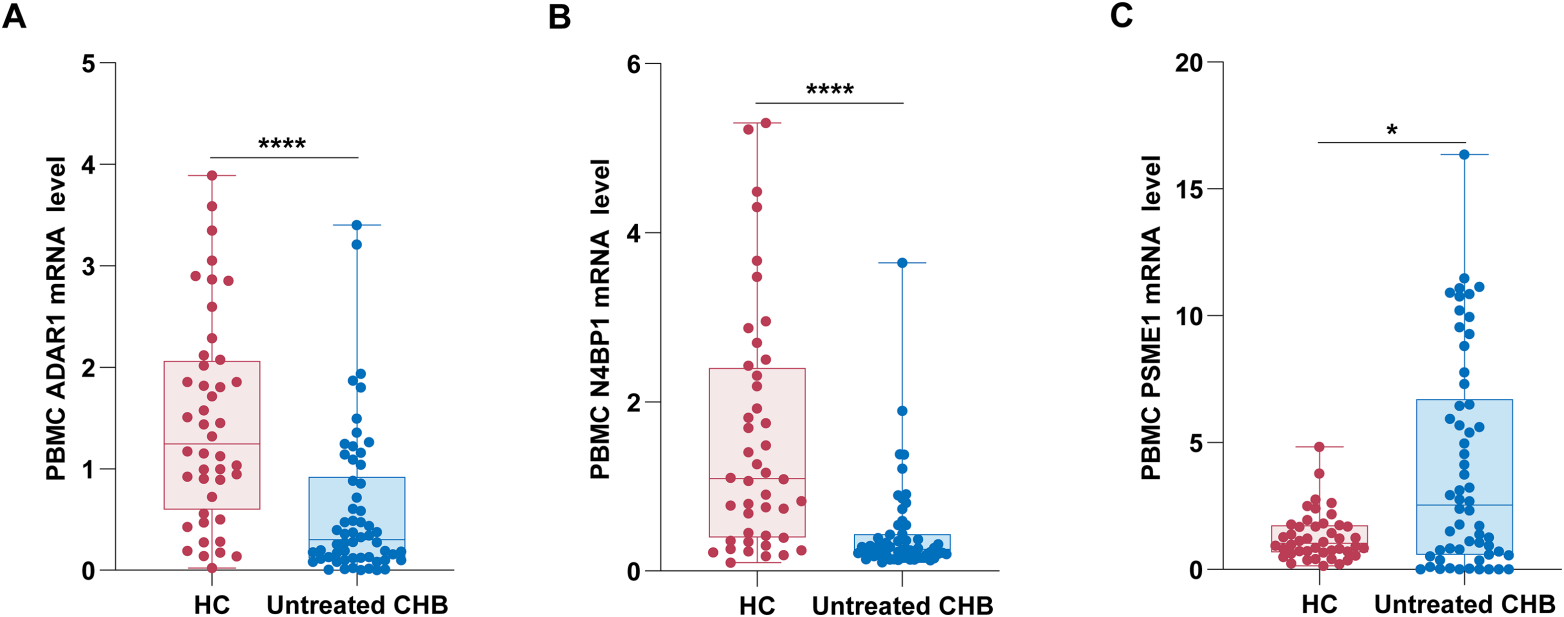
ADAR1, N4BP1, and PSME1 mRNA expressions in PBMCs of untreated CHB patients and healthy controls. ADAR1 **(A)**, N4BP1 **(B)**, and PSME1 **(C)** mRNA levels in PBMCs were analyzed by qRT-PCR. The expression levels were calculated using the 2^-ΔΔct method (Livak method), with GAPDH as the reference gene. *P < 0.05, ****P < 0.0001.

To elucidate the roles of ADAR1, N4BP1, and PSME1 in HBV infection, we assessed the mRNA levels of these ISGs in PBMCs obtained from healthy controls and untreated CHB patients using qRT-PCR. Our analysis demonstrated a marked decrease in the mRNA levels of ADAR1 (P < 0.0001; **Figure 1A**) and N4BP1 (P < 0.0001; **Figure 1B**) in the PBMCs of untreated CHB patients relative to healthy controls. In contrast, we observed a statistically significant increase in PSME1 mRNA levels (P = 0.0119; **Figure 1C**) in PBMCs from untreated CHB patients compared to those from healthy controls. These findings suggest that HBV infection may affect the transcriptional regulation of ADAR1, N4BP1, and PSME1 in PBMCs.

### Characteristics comparison between patients with or without virological or serological response during Peg-IFN-α treatment

Based on the observed changes in HBV DNA and HBsAg levels at week 48 during therapy with Peg-IFN-α, patients were classified into two groups: the VR group and the NVR group. Compared to the NVR group, the VR group exhibited significantly lower HBsAg levels at weeks 0, 12, and 24 (P = 0.0033, P < 0.0001, and P < 0.0001, respectively; as illustrated in **Table 2**; **Supplementary Figure 2**). Notably, the VR group demonstrated significantly lower HBV DNA levels at week 0 compared to the NVR group (P = 0.0257; **Table 2** and **Supplementary Figure 2**), with no significant differences observed at subsequent time points. Additionally, ALT, AST, TBil, PLT, and WBC levels did not demonstrate statistically significant differences between the two groups (**Table 2** and **Supplementary Figure 2**). Furthermore, the baseline HBV genotype distribution exhibited no significant difference between the VR and NVR groups (P = 0.8526; **Supplementary Table 5**).

**Table 2:** Comparison of clinical characteristics between virological response (VR) group and non-virological response (NVR) group.

Characteristics	Time point	VR group (n=43)	NVR group (n=48)	P value
HBsAg (log10 IU/mL)	Week 0	2.385 (1.599, 3.263)	3.194 (2.831, 3.519)	**0.0033**
	Week 12	2.031 (0.8209, 2.789)	3.048 (2.578, 3.441)	**< 0.0001**
	Week 24	1.327 (-0.4685, 2.407)	2.964 (2.332, 3.421)	**< 0.0001**
HBV DNA (log10 IU/mL)	Week 0	1.699 (1.699, 2.512)	1.699 (2.004, 3.000)	**0.0257**
	Week 12	1.699 (1.699, 1.699)	1.699 (1.699, 2.000)	0.1787
	Week 24	1.699 (1.699, 1.699)	1.699 (1.699, 1.699)	0.0929
ALT (U/L)	Week 0	26.00 (16.00, 42.00)	30.00 (19.25, 38.75)	0.4203
	Week 12	25.00 (17.00, 48.00)	35.00 (17.00, 47.75)	0.7202
	Week 24	28.00 (20.00, 43.00)	31.50 (18.00, 58.00)	0.9511
AST (U/L)	Week 0	24.00 (21.00, 36.00)	26.50 (19.25, 35.75)	0.9322
	Week 12	27.00 (22.00, 42.00)	27.00 (22.00, 43.75)	0.8976
	Week 24	29.00 (25.00, 38.00)	27.50 (20.00, 38.75)	0.3093
PLT (10^9/L)	Week 0	154.0 (115.0, 192.0)	149.5 ± 50.44	0.5755
	Week 12	145.3 ± 47.66	146.6 ± 60.17	0.7682
	Week 24	125.0 (96.00, 177.0)	127.0 (97.00, 188.0)	0.7130
WBC (10^9/L)	Week 0	4.259 ± 1.434	4.581 ± 1.397	0.2767
	Week 12	3.990 (3.200, 4.880)	4.453 ± 1.468	0.3058
	Week 24	3.940 (3.000, 4.400)	4.560 (2.980, 5.900)	0.0813

VR, virological response; NVR, non-virological response; HBsAg, hepatitis B surface antigen; ALT, alanine aminotransferase; AST, aspartate aminotransferase; WBC, white blood cells; PLT, platelet. Data are presented as median (interquartile range) or mean ± standard deviation. Intergroup comparisons at each time point were performed using the independent samples t-test for normally distributed data or the Mann-Whitney U test for non-normally distributed data. Bold values indicate statistical significance (P < 0.05).

For HBeAg-negative chronic HBV patients receiving Peg-IFN-α therapy, participants were further categorized into the SR group and NSR group based on the clearance of serum HBsAg or seroconversion after 48 weeks of therapy. HBsAg levels were significantly lower in the SR group at weeks 0, 12, and 24 (P < 0.0001 for all time points; **Table 3**; **Supplementary Figure 3**). The SR group displayed significantly lower HBV DNA levels at weeks 12 and 24 compared to the NSR group (P = 0.0047, and P < 0.0001; as shown in **Table 3** and **Supplementary Figure 3**), with no notable differences at week 0. Furthermore, ALT, AST, and TBil levels did not exhibit significant differences among these groups (**Table 3**; **Supplementary Figure 3**). Notably, WBC levels were significantly lower in the SR group compared to the NSR group at week 24 (P = 0.0081), with no significant disparities observed at weeks 0 and 12 (**Table 3**; **Supplementary Figure 3**). Moreover, the distribution of HBV genotypes was well-balanced between the SR and NSR groups, showing no statistical difference (P = 0.6014; **Supplementary Table 5**).

**Table 3:** Comparison of clinical characteristics between serological response (SR) group and non-serological (NSR) group.

Characteristics	Time point	SR group (n=32)	NSR group (n=59)	P value
HBsAg (log10 IU/mL)	Week 0	1.863 (1.167, 2.678)	3.263 (2.916, 3.574)	**< 0.0001**
	Week 12	1.670 (0.3095, 2.308)	3.056 (2.733, 3.490)	**< 0.0001**
	Week 24	0.6983 (-1.203, 1.834)	2.959 (2.373, 3.430)	**< 0.0001**
HBV DNA (log10 IU/mL)	Week 0	1.699 (1.699, 2.502)	2.000 (1.699, 3.000)	0.4218
	Week 12	1.699 (1.699, 1.699)	1.699 (1.699, 2.000)	**0.0047**
	Week 24	1.699 (1.699, 1.699)	1.699 (1.699, 1.699)	**< 0.0001**
ALT (U/L)	Week 0	25.50 (15.00, 37.75)	30.00 (20.00, 43.00)	0.3526
	Week 12	26.50 (18.00, 47.75)	30.00 (17.00, 48.00)	0.8346
	Week 24	30.50 (21.00, 43.75)	30.00 (18.00, 58.00)	0.8314
AST (U/L)	Week 0	25.00 (21.25, 35.50)	26.00 (20.00, 36.00)	0.7895
	Week 12	27.50 (23.25, 41.75)	27.00 (22.00, 45.00)	0.8931
	Week 24	29.00 (25.00, 38.75)	28.00 (20.00, 38.00)	0.3700
PLT (10^9/L)	Week 0	156.7 ± 52.96	150.3 ± 49.12	0.7237
	Week 12	136.3 ± 44.31	151.2 ± 58.74	0.3379
	Week 24	119.5 (87.75, 171.3)	151.1 ± 64.30	0.2647
WBC (10^9/L)	Week 0	4.239 ± 1.325	4.532 ± 1.464	0.3951
	Week 12	3.780 (3.063, 4.813)	4.491 ± 1.440	0.1013
	Week 24	3.725 (2.815, 4.133)	4.635 ± 1.695	**0.0081**

SR, serological response; NSR, non-serological response; HBsAg, hepatitis B surface antigen; ALT, alanine aminotransferase; AST, aspartate aminotransferase; WBC, white blood cells; PLT, platelet. Data are presented as median (interquartile range) or mean ± standard deviation. Intergroup comparisons at each time point were performed using the independent samples t-test for normally distributed data or the Mann-Whitney U test for non-normally distributed data. Bold values indicate statistical significance (P < 0.05).

### Dynamic changes in ADAR1, N4BP1, and PSME1 mRNA levels during early antiviral therapy in patients treated with Peg-IFN-α

Existing evidence suggests that Peg-IFN-α can increase the expression of ADAR1, N4BP1, and PSME1 in *in vitro* cell culture models. However, it remains unclear how effectively Peg-IFN-α stimulates these genes in PBMCs of HBeAg-negative CHB patients. To investigate the dynamic changes in ADAR1, N4BP1, and PSME1 mRNA levels in PBMCs during early antiviral therapy and their relationship with the early therapeutic response, we measured and analyzed the mRNA expression of these genes in PBMCs.

**Figure 2:**
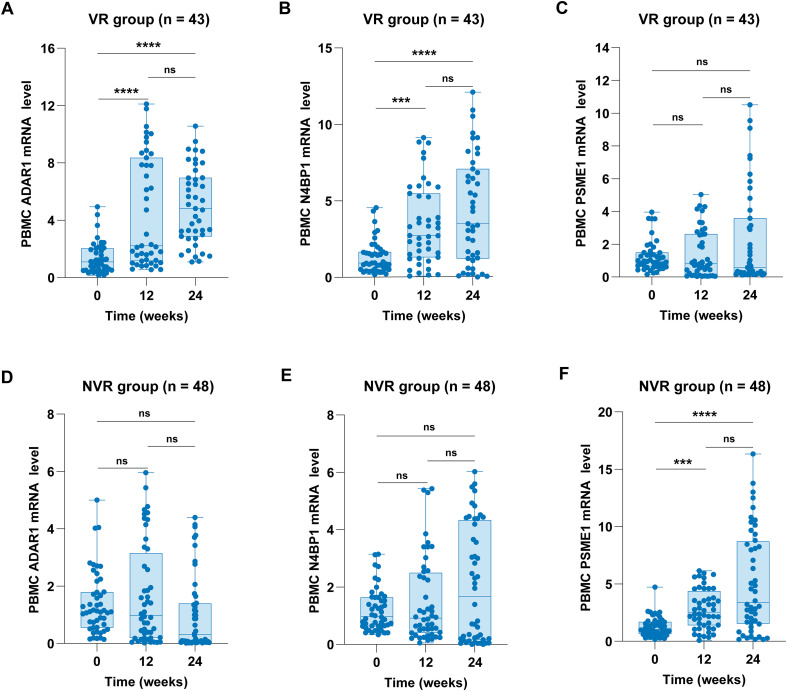
Dynamic changes of ADAR1, N4BP1, and PSME1 mRNA levels in the VR and NVR groups. **(A, D)** ADAR1 mRNA levels in the VR and NVR groups. **(B, E)** N4BP1 mRNA levels in the VR and NVR groups. **(C, F)** PSME1 mRNA levels in the VR and NVR groups. ADAR1, N4BP1, and PSME1 mRNA levels in PBMCs were measured by qRT-PCR. Values are described by median (interquartile range). ***P < 0.001, ****P < 0.0001.

In the VR group, the increase in ADAR1 mRNA levels was statistically significant at both 12 weeks (P < 0.0001) and 24 weeks (P < 0.0001; **Figure 2A**). Similarly, the N4BP1 mRNA levels showed a statistically significant increase at 12 weeks (P = 0.0006) and 24 weeks (P < 0.0001; **Figure 2B**). In contrast, PSME1 mRNA levels did not significantly increase in the VR group at either 12 weeks (P = 0.7351) or 24 weeks (P > 0.9999; **Figure 2C**) following Peg-IFN-α treatment. However, PSME1 mRNA levels were significantly elevated at both 12 weeks (P = 0.0002) and 24 weeks (P < 0.0001; **Figure 2F**) in the NVR group. In addition, no significant change in ADAR1 expression was observed in the NVR group following Peg-IFN-α treatment at 12 weeks (P = 0.5525) or 24 weeks (P = 0.4195; **Figure 2D**). Similarly, Peg-IFN-α did not result in a significant increase in N4BP1 expression in the NVR group at either 12 weeks (P = 0.2092) or 24 weeks (P > 0.9999; **Figure 2E**).

**Figure 3:**
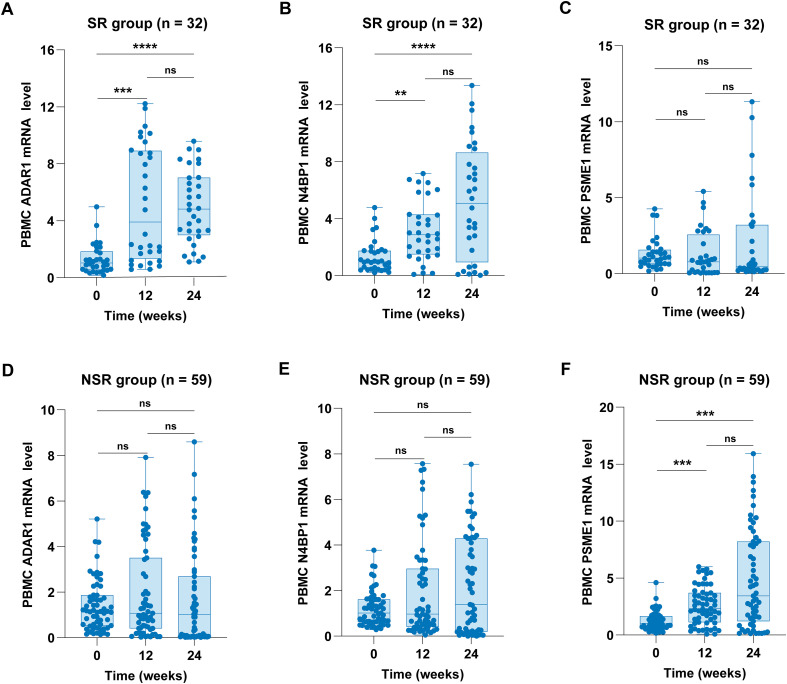
Dynamic changes of ADAR1, N4BP1, and PSME1 mRNA levels in the SR and NSR groups. **(A, D)** ADAR1 mRNA levels in the SR and NSR groups. **(B, E)** N4BP1 mRNA levels in the SR and NSR groups. **(C, F)** PSME1 mRNA levels in the SR and NSR groups. ADAR1, N4BP1, and PSME1 mRNA levels in PBMCs were measured by qRT-PCR. Values are described by median (interquartile range). **P < 0.01, ***P < 0.001, ****P < 0.0001.

Focusing on the serological responders, treatment with Peg-IFN-α in the SR group resulted in a significant increase in ADAR1 expression at both 12 weeks (P = 0.0002) and 24 weeks (P < 0.0001; **Figure 3A**). Similarly, N4BP1 mRNA levels in the SR group increased at both 12 weeks (P = 0.0078) and 24 weeks (P < 0.0001; **Figure 3B**). In contrast, Peg-IFN-α did not lead to a significant increase in PSME1 expression in the SR group at either 12 weeks (P = 0.3072) or 24 weeks (P = 0.5934; **Figure 3C**). However, in the NSR group, Peg-IFN-α treatment successfully increased PSME1 expression at both 12 weeks (P = 0.0003) and 24 weeks (P = 0.0001; **Figure 3F**). Furthermore, no significant change in ADAR1 expression was observed in the NSR group following Peg-IFN-α treatment at 12 weeks (P > 0.9999) or 24 weeks (P = 0.5810; **Figure 3D**). Similarly, Peg-IFN-α did not result in a significant increase in N4BP1 expression in the NSR group at either 12 weeks (P > 0.9999) or 24 weeks (P = 0.8175; **Figure 3E**). Collectively, these findings reveal distinct expression dynamics of ADAR1, N4BP1, and PSME1 mRNA levels between the VR and NVR groups, and between the SR and NSR groups during the early stages of Peg-IFN-α therapy.

### Comparison of the ADAR1, N4BP1, and PSME1 mRNA levels between the VR and NVR groups as well as between the SR and NSR groups

**Figure 4:**
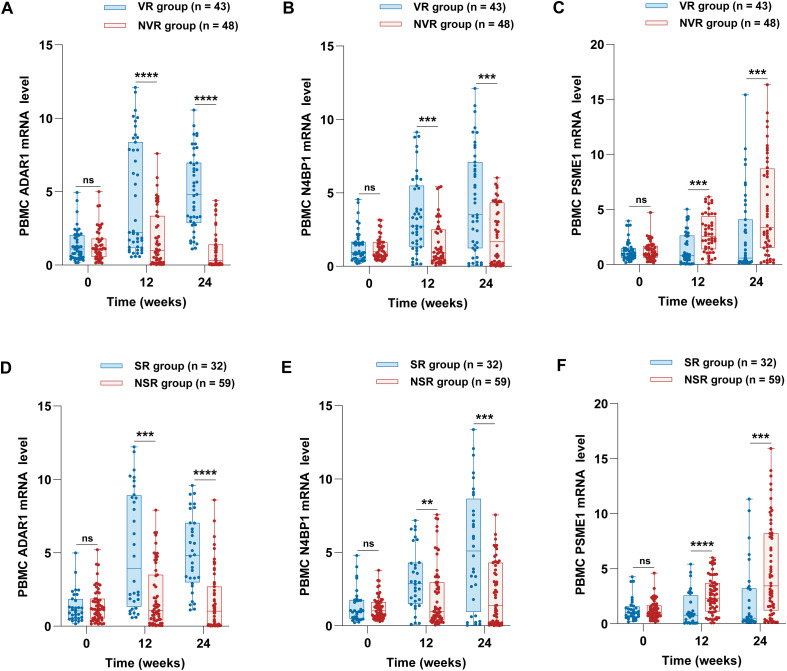
Comparison of the ADAR1, N4BP1, and PSME1 mRNA levels between the responder and non-responder groups. **(A)** ADAR1, **(B)** N4BP1, and **(C)** PSME1 mRNA levels in the VR and NVR groups were compared at weeks 0, 12, and 24. **(D)** ADAR1, **(E)** N4BP1, and **(F)** PSME1mRNA levels in the SR and NSR groups were compared at weeks 0, 12, and 24. ADAR1, N4BP1, and PSME1 mRNA levels in PBMCs were measured by qRT-PCR. Values are described by median (interquartile range). **P < 0.01, ***P < 0.001, ****P < 0.0001.

To further investigate the differences in ADAR1, N4BP1, and PSME1 expression in PBMCs and their potential implications for early treatment response, we compared the mRNA levels of these genes between VR and NVR, as well as between SR and NSR. The results indicated that the mRNA levels of ADAR1 in the VR group were significantly higher than in the NVR group at both 12 weeks (P < 0.0001) and 24 weeks (P < 0.0001; **Figure 4A**). Similarly, the mRNA levels of N4BP1 in the VR group were significantly higher than in the NVR group at both 12 weeks (P = 0.0002) and 24 weeks (P = 0.0006; **Figure 4B**). In contrast, PSME1 mRNA levels were significantly higher in the NVR group than in the VR group at both 12 weeks (P = 0.0001) and 24 weeks (P = 0.0005; **Figure 4C**) of Peg-IFN-α treatment.

We also compared the mRNA levels of ADAR1, N4BP1, and PSME1 between the SR and NSR groups. The mRNA levels of ADAR1 were significantly higher in the SR group compared to the NSR group at both 12 weeks (P = 0.0007) and 24 weeks (P < 0.0001; **Figure 4D**). Similarly, N4BP1 mRNA levels were significantly higher in the SR group at both 12 weeks (P = 0.0040) and 24 weeks (P = 0.0004; **Figure 4E**). Conversely, PSME1 mRNA levels were significantly higher in the NSR group than in the SR group at both 12 weeks (P < 0.0001) and 24 weeks (P = 0.0004; **Figure 4F**) of Peg-IFN-α treatment. Collectively, these findings suggest that patients who achieve a favorable therapeutic response to Peg-IFN-α demonstrate a more rapid increase in ADAR1 and N4BP1 expression, accompanied by a simultaneous decline in PSME1 expression.

### ADAR1, N4BP1, and PSME1 strongly predict patient virological or serological response to Peg-IFN-α therapy

Univariate regression analyses revealed that HBsAg and HBV DNA at week 0, as well as HBsAg, ADAR1, N4BP1, and PSME1 at week 12, along with HBsAg, ADAR1, N4BP1, and PSME1 at week 24 (all P < 0.05), were significantly correlated with VR (**Supplementary Table 6**). Multivariate analyses further identified independent factors associated with VR, specifically HBsAg (aOR = 0.477, P = 0.0047) at week 0; HBsAg (aOR = 0.393, P = 0.0007), ADAR1 (aOR = 1.354, P = 0.0076), N4BP1 (aOR = 1.626, P = 0.0096), and PSME1 (aOR = 0.515, P = 0.0026) at week 12; and HBsAg (aOR = 0.193, P = 0.0140), ADAR1 (aOR = 3.699, P = 0.0003), N4BP1 (aOR = 1.969, P = 0.0146), and PSME1 (aOR = 0.707, P = 0.0463) at week 24 (**Supplementary Table 6**). In terms of predicting SR, univariate analyses identified HBsAg at week 0, HBsAg, ADAR1, N4BP1, and PSME1 at week 12, and HBsAg, WBC, ADAR1, N4BP1, and PSME1 at week 24 (all P < 0.05) as significant predictors (**Supplementary Table 7**). Multivariate analyses further demonstrated that HBsAg (aOR = 0.215, P = 0.0018) at week 0; HBsAg (aOR = 0.123, P = 0.0005), ADAR1 (aOR = 1.452, P = 0.0078), and PSME1 (aOR = 0.412, P = 0.0030) at week 12; and HBsAg (aOR = 0.009, P = 0.0229), ADAR1 (aOR = 2.892, P = 0.0269), and PSME1 (aOR = 0.617, P = 0.0362) at week 24 were identified as independent predictors (**Supplementary Table 7**).

**Figure 5:**
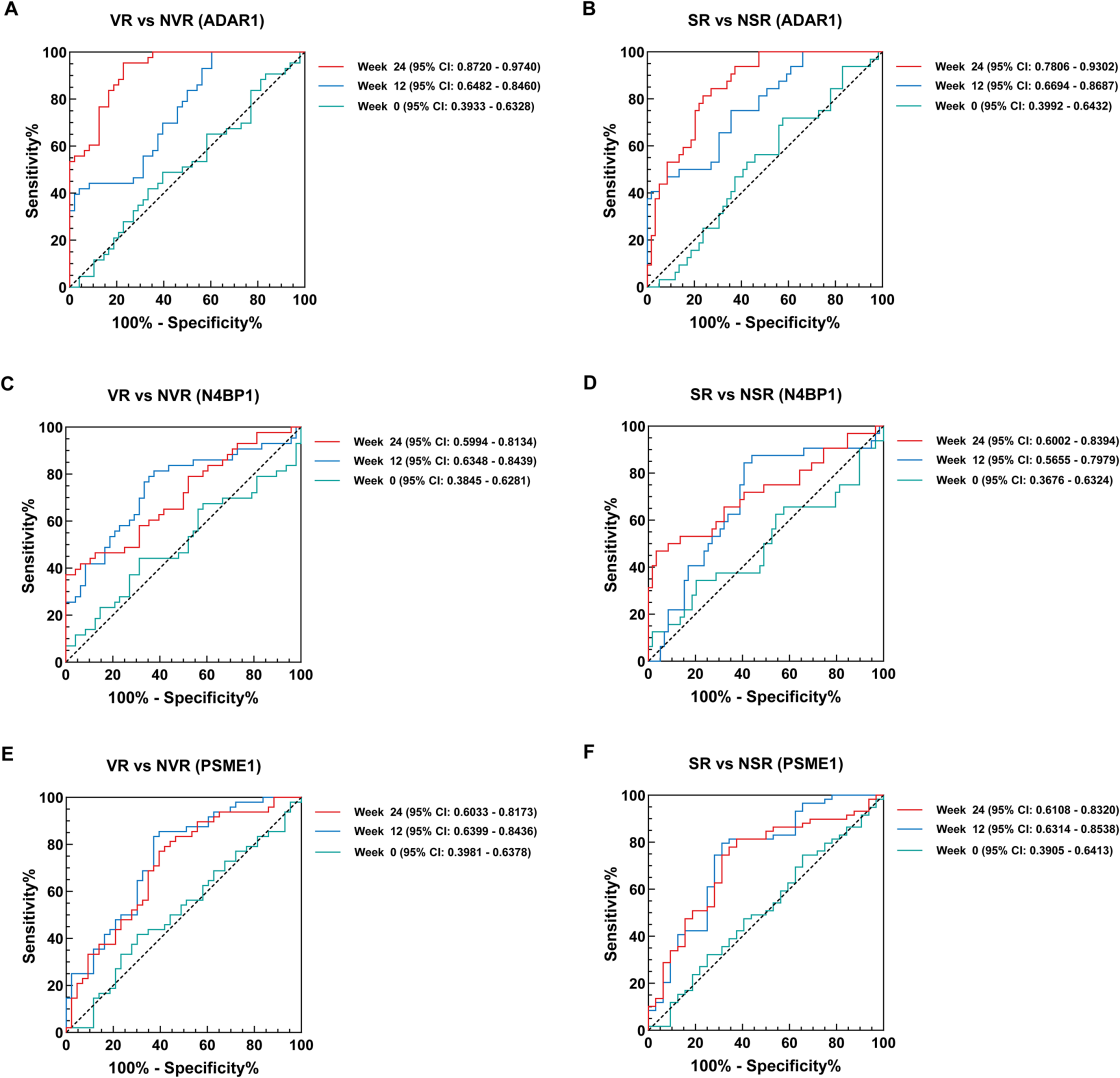
Receiver operating characteristic curves predicting virological and serological response after 48 weeks Peg-IFN-α treatment. **(A, B)** Receiver operating characteristic curves for ADAR1 at weeks 0, 12, and 24 as they predict virological and serological response. **(C, D)** Receiver operating characteristic curves for N4BP1 at weeks 0, 12, and 24 as they predict virological and serological response. **(E, F)** Receiver operating characteristic curves for PSME1 at weeks 0, 12, and 24 as they predict virological and serological response. The Youden index was used to maximize the potential effectiveness of the biomarkers. AUC: area under the curve.

Receiver operating characteristic (ROC) curve analysis further elucidated the predictive utility of ADAR1, N4BP1, and PSME1, particularly at weeks 12 and 24, in predicting VR and SR, as evaluated by the area under the curve (AUC). For VR prediction, ADAR1 at week 24 emerged as the most reliable predictor (AUC = 0.9230, P < 0.0001; **Figure 5A**, **Supplementary Table 8**), with an optimal cutoff value of 1.423. For SR prediction, ADAR1 at week 24 also demonstrated the highest reliability (AUC = 0.8554, P < 0.0001; **Figure 5B**; **Supplementary Table 8**), with an optimal cutoff value of 2.7135. Furthermore, the ROC curves indicated that N4BP1 at week 12 was the most effective predictor for VR (AUC = 0.7393, P < 0.0001; **Figure 5C**; **Supplementary Table 8**), with an optimal cutoff value of 1.1948. Similarly, N4BP1 at week 24 was identified as the most effective predictor for SR (AUC = 0.7198, P = 0.0006; **Figure 5D**; **Supplementary Table 8**), with an optimal cutoff value of 5.9167. Additionally, PSME1 at week 12 was the most effective predictor of VR (AUC = 0.7418, P < 0.0001; **Figure 5E**; **Supplementary Table 8**), with an optimal cutoff value of 1.0834. Similarly, PSME1 at week 24 was the most effective predictor of SR (AUC = 0.7426, P = 0.0001; **Figure 5F**; **Supplementary Table 8**), with an optimal cutoff value of 1.0493.

## Discussion

Currently, Peg-IFN-α is widely utilized in the clinical management of CHB, demonstrating a significantly improved cure rate compared to oral antiviral treatments (You et al., 2023). The primary advantage of Peg-IFN-α lies in its capacity to stimulate host immune responses and reduce the level of intrahepatic covalently closed circular DNA (cccDNA), thereby promoting a more comprehensive viral eradication (Wang et al., 2021). However, a considerable proportion of patients exhibit an inadequate or no response to this therapy, often accompanied by substantial side effects. Consequently, the identification of early predictive biomarkers and the selection of suitable candidates for Peg-IFN-α therapy have emerged as critical research priorities.

Recent studies have indicated that Peg-IFN-α possesses significant antiviral properties, including the enhancement of ISG expression and the modulation of host immune responses (Schoggins et al., 2011). Currently, only a limited number of ISGs, such as SOCS3, STAT1, and MX, have been linked to antiviral effects against HBV, marking them as promising indicators for predicting the clinical efficacy of interferon therapy (Han et al., 2017). Nevertheless, the precise biological functions and antiviral roles of many ISGs remain poorly understood, highlighting the urgent need for straightforward and widely applicable biomarkers to improve clinical outcomes. Our investigation represents a significant advancement in elucidating the role of ISGs in the management of CHB. We demonstrated that ADAR1, N4BP1, and PSME1 may serve as prognostic biomarkers for HBsAg clearance and response to Peg-IFN-α treatment in HBeAg-negative CHB patients. In this study, we explored the dynamic changes in ADAR1, N4BP1, and PSME1 mRNA levels, alongside HBsAg, HBV DNA, ALT, and AST, in HBeAg-negative CHB patients receiving 48 weeks of Peg-IFN-α treatment. Our findings suggest that ADAR1, N4BP1, and PSME1 can provide valuable insights into therapeutic responses, thereby facilitating the development of more personalized treatment regimens. We first examined the mRNA levels of ADAR1, N4BP1, and PSME1 in PBMCs from healthy controls and compared these levels with those in untreated CHB patients. The findings indicated that, in contrast to healthy controls, untreated CHB patients exhibited significantly reduced mRNA levels of ADAR1 and N4BP1, while PSME1 mRNA levels were notably elevated. This phenomenon may be associated with HBV infection, suggesting that chronic HBV infection could lead to the corresponding reduction of ADAR1 and N4BP1 and the elevation of PSME1. Consequently, these ISGs may serve as potential molecular markers for predicting the therapeutic efficacy of interferon.

Recent studies suggest that lower levels of HBsAg during Peg-IFN-α treatment correlate with improved antiviral response rates post-treatment (Marcellin et al., 2013). Furthermore, the loss of HBsAg is linked to a decreased risk of developing cirrhosis and transitioning to HCC, which is a desirable outcome in the management of CHB (Park et al., 2021; Lee et al., 2025; Anderson et al., 2021). In our study, among 91 patients who received 48 weeks of Peg-IFN-α treatment, 32 (35.16%) exhibited a serological response, while 43 (47.25%) achieved a virological response. This response rate aligns with previous research demonstrating the efficacy of Peg-IFN-α therapy (Huang et al., 2017). We further monitored the dynamic changes in serum HBsAg, HBV DNA, ALT, and AST levels at various intervals during the treatment period. HBsAg levels gradually decreased over time, underscoring the challenges associated with the complete clearance of HBsAg, as HBsAg is partially indicative of intrahepatic cccDNA levels (Chan et al., 2007). The stability and transcriptional activity of cccDNA in the liver contribute to its persistence, which complicates the complete elimination of HBV (Gao et al., 2017). Our findings demonstrated that patients responding to Peg-IFN-α therapy exhibited a more pronounced decline in HBsAg and HBV DNA levels during treatment compared to non-responders, underscoring the therapeutic efficacy of Peg-IFN-α in CHB management and indicating that the patient’s immune system begins to respond effectively to HBV. Additionally, a transient decrease in WBC levels at week 24 was observed, attributed to reversible bone marrow suppression caused by interferon, one of the most common adverse reactions of interferon therapy. This decline in WBC is usually dose-related and reversible, with most patients recovering during or after treatment. Despite these potential adverse effects, the administration of Peg-IFN-α in our specific cohort of HBeAg-negative patients, characterized by low HBV DNA levels and normal liver function, was implemented as a proactive, functional cure-oriented strategy. This approach aligns with recent expert consensuses emphasizing early intervention in these patients to maximize the probability of clinical cure (Chen et al., 2019; You et al., 2023).

Prior studies have established a robust association between the expression of ISGs and antiviral immune activation (Li et al., 2015; Mitra et al., 2019; Hou et al., 2014; Fletcher et al., 2015). Consequently, the ability to predict treatment response could enhance Peg-IFN-α therapy by minimizing unnecessary exposure for patients unlikely to benefit. We hypothesized that Peg-IFN-α administration would modulate the mRNA expression of ADAR1, N4BP1, and PSME1 in PBMCs from HBeAg-negative CHB patients, correlating with therapeutic efficacy. As anticipated, our findings revealed differential expression patterns of ADAR1, N4BP1, and PSME1 mRNA in PBMCs of VR and NVR during the initial phase of Peg-IFN-α therapy. In the VR and SR groups, ADAR1 and N4BP1 mRNA levels were significantly increased, while PSME1 mRNA levels were notably elevated in the NVR and NSR groups. Importantly, the mRNA levels of ADAR1 and N4BP1 were significantly higher in the VR group compared to the NVR group, especially after 12 and 24 weeks of treatment. Conversely, the mRNA levels of PSME1 were notably higher in the NVR group than in the VR group during the same time intervals. These results suggest that the transcriptional activity and activation status of these ISGs may serve as prognostic biomarkers for antiviral efficacy and therapeutic outcomes in clinical settings. Therefore, ADAR1 and N4BP1 may actively mediate a potent interferon-induced antiviral response. In contrast, PSME1 may contribute to the negative regulation of ISGs, leading to Peg-IFN-α non-response or resistance. These data suggest that ADAR1, N4BP1, and PSME1 could serve as reliable markers to reflect antiviral efficacy and treatment outcomes during Peg-IFN-α therapy.

Univariate and multivariate regression analyses consistently revealed the predictive value of ADAR1, N4BP1, and PSME1 in the SR and VR prediction at weeks 12 and 24. In this study, ADAR1 and PSME1 mRNA levels at weeks 12 and 24 emerged as independent predictors of SR and VR in both univariate and multivariate analyses, while N4BP1 expression levels were independently associated with VR at these time points. Notably, univariate and multivariate models identified HBsAg as significantly correlated with both VR and SR at weeks 0, 12, and 24. In clinical practice, early on-treatment HBsAg levels are recognized milestones for deciding whether to continue interferon therapy. By incorporating on-treatment changes in HBsAg levels into the prediction model, we aimed to simulate real-world response-guided treatment strategies. More importantly, including HBsAg in the multivariable model allowed us to assess the incremental predictive value of the identified gene biomarkers. After adjustment for HBsAg levels, the mRNA expression levels of ADAR1, N4BP1, and PSME1 remained independently associated with both VR and SR. This independence suggests that these ISGs provide distinct biological insights into host immune status that cannot be fully captured by viral protein levels alone. Furthermore, the ROC curve analysis revealed that N4BP1 mRNA levels possessed the highest predictive accuracy for VR at week 12 and for SR at week 24, and PSME1 mRNA levels possessed the highest predictive accuracy for VR and SR at week 12. Moreover, ADAR1 demonstrated optimal predictive value for VR and SR at week 24. These findings suggest that the mRNA levels of ADAR1, N4BP1, and PSME1 in PBMCs of HBeAg-negative CHB patients during initial Peg-IFN-α therapy may serve as biomarkers for treatment response and prognosis. While HBsAg levels exhibited significant predictive ability in our study, we believe that ADAR1, N4BP1, and PSME1 possess potential value for further research and broader application. The combination of these biomarkers may offer a more comprehensive approach to predicting treatment outcomes, particularly in cases where HBsAg alone may not fully reflect the underlying immune dynamics or viral persistence. Additionally, in clinical practice, ADAR1, N4BP1, and PSME1 may enhance the predictive ability of HBsAg.

However, several limitations exist in this study. First, ADAR1, N4BP1, and PSME1 were selected primarily due to their distinct anti-HBV mechanisms and the lack of clinical studies evaluating their predictive value for interferon therapy in CHB patients, rather than being identified through an unbiased transcriptomic or whole genome screening approach. Therefore, the potential predictive value of other unexamined ISGs remains to be fully explored. Second, our enrolled patients were mainly HBeAg-negative individuals with low HBV DNA levels and normal liver function, which implies that our findings may not be directly generalizable to broader CHB populations. Third, the follow-up period ended at week 48 during ongoing Peg-IFN-α treatment, and no post-treatment data were available. Consequently, we could not evaluate whether the dynamic changes in ADAR1, N4BP1, and PSME1 mRNA levels predict sustained virological or serological response, or functional cure after treatment discontinuation, which represents the most clinically relevant outcome of Peg-IFN-α therapy. Fourth, regarding our prediction models, utilizing the early on-treatment decline of HBsAg as a predictor for a composite virological response endpoint that includes HBsAg criteria introduces a certain degree of definitional overlap, although this approach was intended to simulate response-guided clinical practice in the real-world. Fifth, because gene expression was measured in PBMCs, we cannot entirely exclude the possibility that subtle changes in the proportions of lymphocytes or monocytes within the PBMCs fraction may have partially influenced the observed differences in mRNA expression levels, although PBMCs isolation by density gradient centrifugation (Ficoll) largely removes granulocytes. Finally, the sample size was relatively small and predominantly consisted of patients infected with HBV genotypes B or C. Future studies with larger cohorts, extended off-treatment follow-up, and approaches such as cell sorting or single-cell RNA sequencing will be important to validate these findings, clarify effects specific to different cell types, and determine whether these biomarkers can reliably identify patients likely to achieve a durable response.

## Conclusion

These findings provide valuable insights for clinical practice, indicating that ADAR1, N4BP1, and PSME1 in the PBMCs of HBeAg-negative CHB patients during the initial stages of Peg-IFN-α treatment may correlate with treatment outcomes and HBsAg clearance. This suggests that ADAR1, N4BP1, and PSME1 have the potential to serve as effective biomarkers for enhancing clinical treatment responses. Furthermore, monitoring the levels of ADAR1, N4BP1, and PSME1 can facilitate timely adjustments to treatment strategies, thereby improving treatment success rates.

## Data Availability

The raw data supporting the conclusions of this article will be made available by the authors, without undue reservation.
