# Supplementary figures and images for: Altered expression of ADAR1, N4BP1, and PSME1 in PBMCs correlated with therapeutic outcomes in HBeAg-negative chronic hepatitis B patients treated with Peg-IFN-α
Source: Front Cell Infect Microbiol. 2026 Apr 13;16:1749013. doi: 10.3389/fcimb.2026.1749013 (PMC13111010; doi:10.3389/fcimb.2026.1749013)

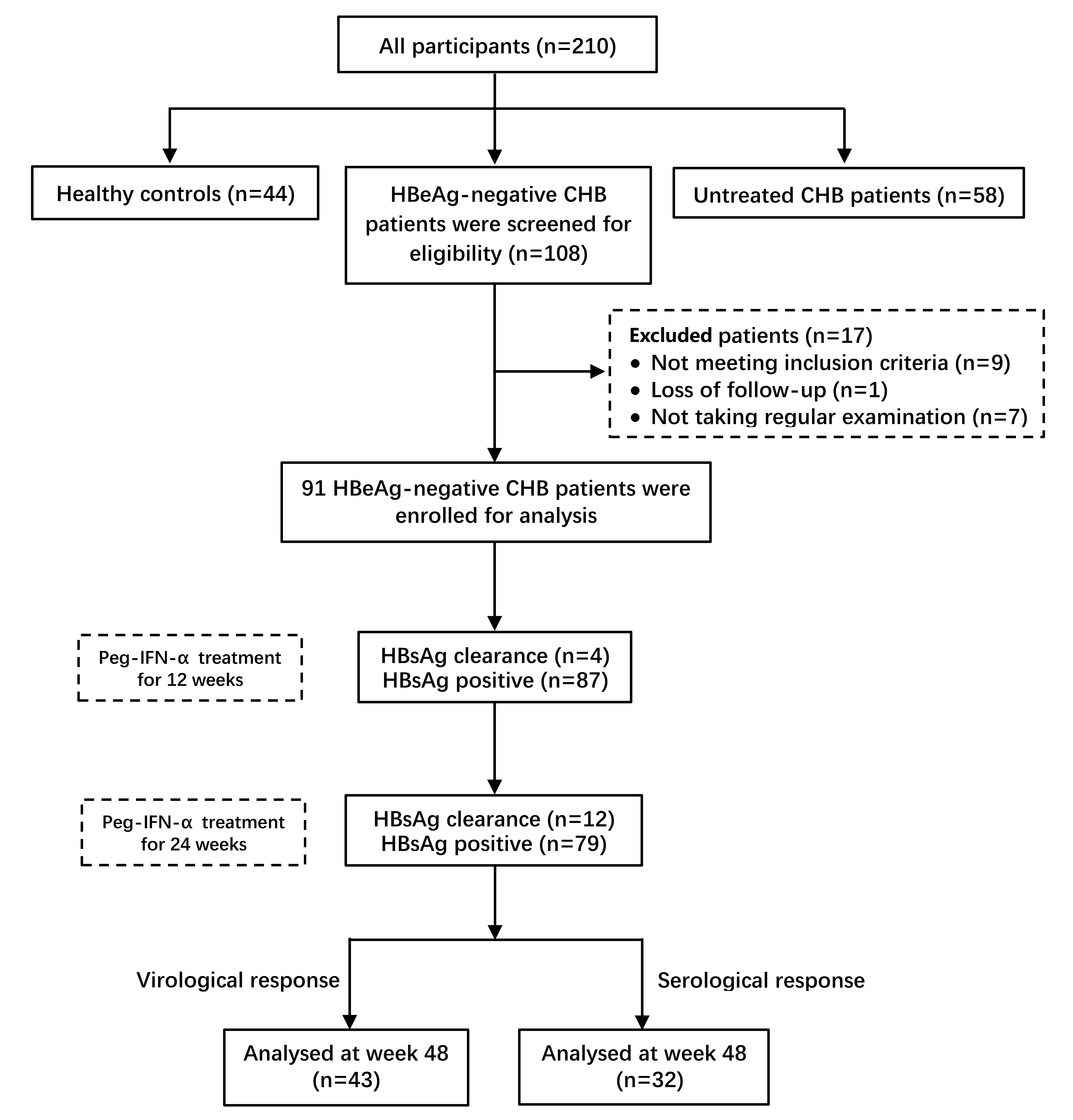

Supplement: Supplementary file 1 [file Image1.tif]
