# Supplementary material for: Altered expression of ADAR1, N4BP1, and PSME1 in PBMCs correlated with therapeutic outcomes in HBeAg-negative chronic hepatitis B patients treated with Peg-IFN-α
Source: Front Cell Infect Microbiol. 2026 Apr 13;16:1749013. doi: 10.3389/fcimb.2026.1749013 (PMC13111010; doi:10.3389/fcimb.2026.1749013)
Supplement: Supplementary file 4 [file Table1.docx]

| Table S1 A priori sample size calculation for all cohorts. | | | | | | |
| --- | --- | --- | --- | --- | --- | --- |
| Statistical analysis model | Assumed effect size | α | Power (1-β) | Allocation ratio | Minimum required sample size | Actual enrolled sample size |
| Two-group comparison (VR vs NVR) | Cohen's d = 0.65 | 0.05 | 0.80 | 0.80 | total n = 78  (VR = 35, NVR = 43) | total n = 91  (VR = 43, NVR = 48) |
| Two-group comparison (SR vs. NSR) | Cohen's d = 0.65 | 0.05 | 0.80 | 0.50 | total n = 86  (SR=29, NSR = 57) | total n = 91  (SR = 32, NSR = 59) |
| Two-group comparison (HC vs. Untreated CHB) | Cohen's d = 0.65 | 0.05 | 0.80 | 0.60 | total n = 82  (HC = 31, Untreated CHB = 51) | total n = 102  (HC = 44, Untreated CHB = 58) |
| Repeated measures (weeks 0, 12, and 24) | Cohen's f = 0.30 | 0.05 | 0.80 | - | total n = 20 | VR = 43, NVR = 48; SR = 32, NSR = 59 (All subgroups > 20) |
| VR, virological response; NVR, non-virological response; SR, serological response; NSR, non-serological response; HC, Healthy controls; Sample size was calculated a priori using G*Power software (version 3.1.9.7). A medium effect size of Cohen's f = 0.30 was assumed for repeated measures comparisons, and Cohen's d = 0.65 for between-group comparisons. Two-sided α = 0.05 and minimum power of 80% were applied. | | | | | | |
