# Supplementary material for: Altered expression of ADAR1, N4BP1, and PSME1 in PBMCs correlated with therapeutic outcomes in HBeAg-negative chronic hepatitis B patients treated with Peg-IFN-α
Source: Front Cell Infect Microbiol. 2026 Apr 13;16:1749013. doi: 10.3389/fcimb.2026.1749013 (PMC13111010; doi:10.3389/fcimb.2026.1749013)
Supplement: Supplementary file 5 [file Table2.docx]

| **Table S2** Primer sequences used in this study | |
| --- | --- |
| Primers sequence(5’-3’) | |
| GAPDH RP | 5’-TGACACGTTGGCAGTGG-3’ |
| GAPDH FP | 5’-GGGGCTCTCCAGAACATC-3’ |
| ADAR1 RP | 5’- TGCAGGAGGTGGTCAA-3’ |
| ADAR1 FP | 5’- CAAGGTGGAGAACGGAGA-3’ |
| N4BP1 RP | 5’- TCTCAGTCGCTTTCACTT-3’ |
| N4BP1 FP | 5’-TCCGTGAGTTTGTCTTTTC-3’ |
| PSME1 RP | 5’- CACGAAACACATCCACCTT-3’ |
| PSME1 FP | 5’- TGCCCACTCCACTCCTT-3’ |
