# Supplementary material for: Altered expression of ADAR1, N4BP1, and PSME1 in PBMCs correlated with therapeutic outcomes in HBeAg-negative chronic hepatitis B patients treated with Peg-IFN-α
Source: Front Cell Infect Microbiol. 2026 Apr 13;16:1749013. doi: 10.3389/fcimb.2026.1749013 (PMC13111010; doi:10.3389/fcimb.2026.1749013)
Supplement: Supplementary file 6 [file Table3.docx]

| **Table S3** Serological response during Peg-IFN-α treatment | | | | |
| --- | --- | --- | --- | --- |
| Serological response(n, %) | 12w | 24w | 36w | 48w |
| HBsAg clearance | 4(4.40%) | 12(13.19%) | 20(21.98%) | 32(35.16%) |
| HBsAg seroconversion | 0(0.00%) | 4(4.40%) | 10(10.99%) | 18(19.78%) |
