# Supplementary material for: Altered expression of ADAR1, N4BP1, and PSME1 in PBMCs correlated with therapeutic outcomes in HBeAg-negative chronic hepatitis B patients treated with Peg-IFN-α
Source: Front Cell Infect Microbiol. 2026 Apr 13;16:1749013. doi: 10.3389/fcimb.2026.1749013 (PMC13111010; doi:10.3389/fcimb.2026.1749013)
Supplement: Supplementary file 7 [file Table4.docx]

| **Table S4** Virological response during Peg-IFN-α treatment | | | | |
| --- | --- | --- | --- | --- |
| Virological response (n, %) | 12w | 24w | 36w | 48w |
| HBsAg decreased by >1 log10 IU/ml | 6(6.59%) | 21(23.08%) | 34(37.36%) | 43(47.25%) |
| HBV DNA decreased by >2 log10 IU/ml | 0 (0.00%) | 0 (0.00%) | 0 (0.00%) | 0 (0.00%) |
