# Supplementary material for: Altered expression of ADAR1, N4BP1, and PSME1 in PBMCs correlated with therapeutic outcomes in HBeAg-negative chronic hepatitis B patients treated with Peg-IFN-α
Source: Front Cell Infect Microbiol. 2026 Apr 13;16:1749013. doi: 10.3389/fcimb.2026.1749013 (PMC13111010; doi:10.3389/fcimb.2026.1749013)
Supplement: Supplementary file 8 [file Table5.docx]

| **Table S5** Comparison of baseline HBV genotype distribution between response and non-response groups. | | | | | | | |
| --- | --- | --- | --- | --- | --- | --- | --- |
| Characteristics | All (n=91) | VR group (n=43) | NVR group (n=48) | P value | SR group  (n=32) | NSR group  (n=59) | P value |
| HBV genotype, n (%) |  |  |  | 0.8526 |  |  | 0.6014 |
| Genotype B | 54 (59.3) | 26 (60.5) | 28 (58.3) |  | 20 (62.5) | 34 (57.6) |  |
| Genotype C | 33 (36.3) | 15 (34.9) | 18 (37.5) |  | 10 (31.3) | 23 (39.0) |  |
| Others | 4 (4.4) | 2 (4.7) | 2 (4.2) |  | 2 (6.3) | 2 (3.4) |  |
| Data are expressed as number (%); VR, virological response; NVR, non-virological response; SR, serological response; NSR, non-serological response. | | | | | | | |
