# Supplementary material for: Altered expression of ADAR1, N4BP1, and PSME1 in PBMCs correlated with therapeutic outcomes in HBeAg-negative chronic hepatitis B patients treated with Peg-IFN-α
Source: Front Cell Infect Microbiol. 2026 Apr 13;16:1749013. doi: 10.3389/fcimb.2026.1749013 (PMC13111010; doi:10.3389/fcimb.2026.1749013)
Supplement: Supplementary file 9 [file Table6.docx]

| **Table S6** On-treatment variables associated with virological response according to univariate and multivariate analyses. | | | | | | | | | | | | |
| --- | --- | --- | --- | --- | --- | --- | --- | --- | --- | --- | --- | --- |
| Variables | Univariate analyses |  | Multivariate analyses |  | Univariate analyses |  | Multivariate analyses |  | Univariate analyses |  | Multivariate analyses |  |
|  | OR(95%CI) | P-value | aOR(95%CI) | P-value | OR(95%CI) | P- value | aOR(95%CI) | P-value | OR(95%CI) | P-value | aOR(95%CI) | P-  value |
|  | **Week0** |  |  |  | **Week12** |  |  |  | **Week24** |  |  |  |
| Gender | 0.860(0.376, 1.966) | 0.7216 |  |  | 0.860(0.376, 1.966) | 0.7216 |  |  | 0.860(0.376, 1.966) | 0.7216 |  |  |
| Age | 1.004(0.962, 1.047) | 0.8509 |  |  | 1.004(0.962, 1.047) | 0.8509 |  |  | 1.004(0.962, 1.047) | 0.8509 |  |  |
| HBsAg | 0.484(0.303, 0.774) | **0.0024** | 0.477(0.286,0.797) | **0.0047** | 0.446(0.285,0.698) | **0.0004** | 0.393(0.229,0.676) | **0.0007** | 0.354(0.220,0.568) | **0.0017** | 0.193(0.051,0.717) | **0.0140** |
| HBV DNA | 0.507(0.264, 0.972) | **0.0408** | 0.665(0.3271,1.3539) | 0.2611 | 0.874(0.337,2.267) | 0.7823 |  |  | 0.518(0.059,4.529) | 0.5529 |  |  |
| ALT | 0.993(0.977, 1.008) | 0.3919 |  |  | 1.002(0.988,1.017) | 0.7006 |  |  | 0.998(0.982,1.015) | 0.8945 |  |  |
| AST | 0.991(0.963, 1.021) | 0.5847 |  |  | 1.003(0.988,1.018) | 0.6642 |  |  | 1.009(0.989,1.029) | 0.3698 |  |  |
| WBC | 1.004(0.961, 1.048) | 0.8527 |  |  | 0.888(0.668,1.180) | 0.4136 |  |  | 0.794(0.610,1.034) | 0.0876 |  |  |
| TBil | 1.019(0.927, 1.120) | 0.6886 |  |  | 1.004(0.920,1.095) | 0.9190 |  |  | 0.990(0.902,1.087) | 0.8420 |  |  |
| PLT | 1.002(0.994, 1.010) | 0.5341 |  |  | 0.999(0.991,1.007) | 0.9053 |  |  | 0.997(0.991,1.004) | 0.5264 |  |  |

**Continued Table S6** On-treatment variables associated with virological response according to univariate and multivariate analyses.

| Variables | Univariate analyses |  | Multivariate analyses |  | Univariate analyses |  | Multivariate analyses |  | Univariate analyses |  | Multivariate analyses |  |
| --- | --- | --- | --- | --- | --- | --- | --- | --- | --- | --- | --- | --- |
|  | OR(95%CI) | P-value | aOR(95%CI) | P-value | OR(95%CI) | P-value | aOR(95%CI) | P-value | OR(95%CI) | P-value | aOR(95%CI) | P-  value |
| ADAR1 | 0.732(0.489,1.094) | 0.1283 |  |  | 1.361(1.150,1.612) | **0.0003** | 1.354(1.083,1.692) | **0.0076** | 2.698(1.813,4.015) | **0.0001** | 3.699(1.803,7.589) | **0.0003** |
| N4BP1 | 0.922(0.577,1.474) | 0.9811 |  |  | 1.607(1.245,2.075) | **0.0007** | 1.626(1.125,2.351) | **0.0096** | 1.338(1.131,1.583) | **0.0028** | 1.969(1.142,3.394) | **0.0146** |
| PSME1 | 0.904(0.567,1.443) | 0.6750 |  |  | 0.593(0.443,0.794) | **0.0044** | 0.515(0.335,0.793) | **0.0026** | 0.848(0.755,0.954) | **0.0059** | 0.707(0.503,0.994) | **0.0463** |
| Values expressed as odds ratio (OR) and 95% confidence interval (CI); aOR, adjusted odds ratio; ADAR1, adenosine deaminase acting on RNA 1; N4BP1, NEDD4-binding protein 1; PSME1, proteasome activator complex subunit 1; HBsAg, hepatitis B surface antigen; ALT, alanine aminotransferase; AST, aspartate aminotransferase; WBC, white blood cells; TBil: total bilirubin; PLT: platelet. Bold values are statistically significant, P < 0.05. | | | | | | | | | | | | |
