# Supplementary material for: Altered expression of ADAR1, N4BP1, and PSME1 in PBMCs correlated with therapeutic outcomes in HBeAg-negative chronic hepatitis B patients treated with Peg-IFN-α
Source: Front Cell Infect Microbiol. 2026 Apr 13;16:1749013. doi: 10.3389/fcimb.2026.1749013 (PMC13111010; doi:10.3389/fcimb.2026.1749013)
Supplement: Supplementary file 10 [file Table7.docx]

| **Table S7** On-treatment variables associated with serological response according to univariate and multivariate analyses. | | | | | | | | | | | | |
| --- | --- | --- | --- | --- | --- | --- | --- | --- | --- | --- | --- | --- |
| Variables | Univariate analyses |  | Multivariate analyses |  | Univariate analyses |  | Multivariate analyses |  | Univariate analyses |  | Multivariate analyses |  |
|  | OR(95%CI) | P-value | aOR(95%CI) | P-value | OR(95%CI) | P- value | aOR(95%CI) | P-value | OR(95%CI) | P-value | aOR(95%CI) | P-  value |
|  | **Week0** |  |  |  | **Week12** |  |  |  | **Week24** |  |  |  |
| Gender | 0.580(0.240, 1.396) | 0.2011 |  |  | 0.580(0.240, 1.396) | 0.2011 |  |  | 0.580(0.240, 1.396) | 0.2011 |  |  |
| Age | 1.009(0.965, 1.056) | 0.6723 |  |  | 1.009(0.965, 1.056) | 0.6723 |  |  | 1.009(0.965, 1.056) | 0.6723 |  |  |
| HBsAg | 0.216(0.111, 0.419) | **0.0006** | 0.215(0.106,0.434) | **0.0018** | 0.207(0.105, 0.408) | **0.0005** | 0.123(0.044,0.339) | **0.0005** | 0.152(0.069,0.335) | **0.0003** | 0.009(0.001,0.529) | **0.0229** |
| HBV DNA | 0.496(0.245, 1.004) | 0.0513 |  |  | 0.307(0.081, 1.167) | 0.0832 |  |  | 0.003(0.006, 1.066) | 0.9974 |  |  |
| ALT | 0.988(0.968, 1.007) | 0.2290 |  |  | 1.003(0.988, 1.018) | 0.6737 |  |  | 0.996(0.978,1.014) | 0.6686 |  |  |
| AST | 0.989(0.958, 1.020) | 0.5024 |  |  | 0.995(0.979, 1.012) | 0.6181 |  |  | 1.007(0.987,1.027) | 0.4634 |  |  |
| WBC | 0.860(0.629,1.176) | 0.3467 | 1.028(0.674,1.567) | 0.8967 | 0.805(0.591, 1.096) | 0.1696 |  |  | 0.692(0.509,0.942) | **0.0194** |  |  |
| TBil | 1.019(0.924, 1.124) | 0.6931 |  |  | 1.009(0.921, 1.104) | 0.8452 |  |  | 0.993(0.901,1.095) | 0.8988 |  |  |
| PLT | 1.002(0.993,1.011) | 0.5617 |  |  | 0.994(0.986, 1.002) | 0.2113 |  |  | 0.995(0.988,1.002) | 0.2356 |  |  |

**Continued Table S7** On-treatment variables associated with serological responses according to univariate and multivariate analyses.

| Variables | Univariate analyses |  | Multivariate analyses |  | Univariate analyses |  | Multivariate analyses |  | Univariate analyses |  | Multivariate analyses |  |
| --- | --- | --- | --- | --- | --- | --- | --- | --- | --- | --- | --- | --- |
|  | OR(95%CI) | P-value | aOR(95%CI) | P-value | OR(95%CI) | P-value | aOR(95%CI) | P-value | OR(95%CI) | P-value | aOR(95%CI) | P-  value |
| ADAR1 | 0.706(0.450,1.107) | 0.1295 |  |  | 1.390(1.181,1.636) | **0.0073** | 1.452(1.102,1.912) | **0.0078** | 1.729(1.374,2.176) | **0.0003** | 2.892(1.128,7.410) | **0.0269** |
| N4BP1 | 0.752(0.445,1.271) | 0.4993 |  |  | 1.200(0.983,1.465) | **0.0019** | 1.043(0.777,1.399) | 0.7761 | 1.385(1.170,1.639) | **0.0001** | 1.431(0.771,2.656) | 0.2559 |
| PSME1 | 0.804(0.480,1.348) | 0.4097 |  |  | 0.583(0.420,0.810) | **0.0012** | 0.412(0.229,0.741) | **0.0030** | 0.803(0.691,0.932) | **0.0039** | 0.617(0.392,0.969) | **0.0362** |
| Values expressed as odds ratio (OR) and 95% confidence interval (CI); aOR, adjusted odds ratio; ADAR1, adenosine deaminase acting on RNA 1; N4BP1, NEDD4-binding protein 1; PSME1, proteasome activator complex subunit 1; HBsAg, hepatitis B surface antigen; ALT, alanine aminotransferase; AST, aspartate aminotransferase; WBC, white blood cells; TBil: total bilirubin; PLT: platelet. Bold values are statistically significant, P < 0.05. | | | | | | | | | | | | |
