# Supplementary material for: Altered expression of ADAR1, N4BP1, and PSME1 in PBMCs correlated with therapeutic outcomes in HBeAg-negative chronic hepatitis B patients treated with Peg-IFN-α
Source: Front Cell Infect Microbiol. 2026 Apr 13;16:1749013. doi: 10.3389/fcimb.2026.1749013 (PMC13111010; doi:10.3389/fcimb.2026.1749013)
Supplement: Supplementary file 11 [file Table8.docx]

| **Table S8** The predictive performance of ADAR1, N4BP1and PSME1mRNA levels in predicting VR and SR to 48 weeks of Peg-IFN-α treatment. | | | | | | | | | | | | |
| --- | --- | --- | --- | --- | --- | --- | --- | --- | --- | --- | --- | --- |
|  |  | ADAR1 | N4BP1 | PSME1 |  | ADAR1 | N4BP1 | PSME1 |  | ADAR1 | N4BP1 | PSME1 |
| VR prediction |  |  | **Week 0** |  |  |  | **Week12** |  |  |  | **Week24** |  |
|  | AUC | 0.5131 | 0.5063 | 0.5179 |  | 0.7471 | 0.7393 | 0.7418 |  | 0.9230 | 0.7064 | 0.7103 |
|  | (95% CI) | (0.3933 -   0.6328) | (0.3845 -   0.6281) | (0.3981 -   0.6378) |  | (0.6482 -   0.8460) | (0.6348 -   0.8439) | (0.6399 -  0.8436) |  | (0.8720 -   0.9740) | (0.5994 -   0.8134) | (0.6033 -   0.8173) |
|  | Cut-off value | 1.8254 | 1.4144 | 2.6618 |  | 0.5364 | 1.1948 | 1.0834 |  | 1.423 | 6.0701 | 1.4304 |
|  | Sensitivity (%) | 30.20 | 44.20 | 11.60 |  | 100.00 | 81.40 | 83.30 |  | 95.30 | 37.20 | 77.10 |
|  | Specificity (%) | 77.10 | 68.70 | 97.90 |  | 39.60 | 62.50 | 62.80 |  | 77.10 | 100.00 | 60.50 |
|  | P value | 0.8300 | 0.9177 | 0.7687 |  | **< 0.0001** | **< 0.0001** | **< 0.0001** |  | **< 0.0001** | **0.0007** | **0.0006** |
| SR prediction |  |  | **Week 0** |  |  |  | **Week12** |  |  |  | **Week24** |  |
|  | AUC | 0.5212 | 0.5000 | 0.5159 |  | 0.7691 | 0.6817 | 0.7426 |  | 0.8554 | 0.7198 | 0.7214 |
|  | (95% CI) | (0.3992 -   0.6432) | (0.3676 -   0.6324) | (0.3905 - 0.6413) |  | \| (0.6694 -  0.8687) \| \| --- \| | (0.5655 -   0.7979) | (0.6314 -   0.8538) |  | (0.7806 -   0.9302) | (0.6002 -   0.8394) | (0.6108 -   0.8320) |
|  | Cut-off value | 0.299 | 1.6682 | 3.5208 |  | 1.6897 | 1.3075 | 1.0493 |  | 2.7135 | 5.9167 | 0.7545 |
|  | Sensitivity (%) | 96.90 | 34.40 | 9.40 |  | 75.00 | 84.40 | 79.70 |  | 81.30 | 46.90 | 81.40 |
|  | Specificity (%) | 11.90 | 79.70 | 98.30 |  | 64.40 | 59.30 | 68.70 |  | 76.30 | 96.60 | 62.50 |
|  | P value | 0.7395 | > 0.9999 | 0.8031 |  | **< 0.0001** | **0.0044** | **0.0001** |  | **< 0.0001** | **0.0006** | **0.0005** |
| ADAR1, adenosine deaminase acting on RNA 1; N4BP1, NEDD4-binding protein 1; PSME1, proteasome activator complex subunit 1; AUC, area under the ROC curve; CI, confidence interval; VR, virological response; SR, serological response; Bold values are statistically significant P < 0.05. | | | | | | | | | | | | |
